# Epicardial adipose tissue radiodensity is associated with all-cause mortality in patients undergoing hemodialysis

**DOI:** 10.1038/s41598-021-02427-4

**Published:** 2021-11-29

**Authors:** Seong Soon Kwon, Kyoungjin Choi, Bo Da Nam, Haekyung Lee, Nam-Jun Cho, Byoung Won Park, Hyoungnae Kim, Hyunjin Noh, Jin Seok Jeon, Dong Cheol Han, Sujeong Oh, Soon Hyo Kwon

**Affiliations:** 1 Division of Cardiology, Department of Internal Medicine, Soonchunhyang University Seoul Hospital, Seoul, Republic of Korea; 2 Division of Nephrology, Department of Internal Medicine, Soonchunhyang University Seoul Hospital, Seoul, Republic of Korea; 3 Department of Radiology, Soonchunhyang University Seoul Hospital, Seoul, Republic of Korea; 4 Division of Nephrology, Department of Internal Medicine, Soonchunhyang University Cheonan Hospital, Cheonan, Republic of Korea

**Keywords:** Cardiovascular biology, Renal replacement therapy

## Abstract

The radiodensity and volume of epicardial adipose tissue (EAT) on computed tomography angiography (CTA) may provide information regarding cardiovascular risk and long-term outcomes. EAT volume is associated with mortality in patients undergoing incident hemodialysis. However, the relationship between EAT radiodensity/volume and all-cause mortality in patients with end-stage renal disease (ESRD) undergoing maintenance hemodialysis remains elusive. In this retrospective study, EAT radiodensity (in Hounsfield units) and volume (in cm^3^) on coronary CTA were quantified for patients with ESRD using automatic, quantitative measurement software between January 2012 and December 2018. All-cause mortality data (up to December 2019) were obtained from the Korean National Statistical Office. The prognostic values of EAT radiodensity and volume for predicting long-term mortality were assessed using multivariable Cox regression models, which were adjusted for potential confounders. A total of 221 patients (mean age: 64.88 ± 11.09 years; 114 women and 107 men) with ESRD were included. The median follow-up duration (interquartile range) after coronary CTA was 29.63 (range 16.67–44.7) months. During follow-up, 82 (37.1%) deaths occurred. In the multivariable analysis, EAT radiodensity (hazard ratio [HR] 1.055; 95% confidence interval [CI] 1.015–1.095; *p* = 0.006) was an independent predictor of all-cause mortality in patients with ESRD. However, EAT volume was not associated with mortality. Higher EAT radiodensity on CTA is associated with higher long-term all-cause mortality in patients undergoing prevalent hemodialysis, highlighting its potential as a prognostic imaging biomarker in patients undergoing hemodialysis.

## Introduction

Epicardial adipose tissue (EAT) is located between the myocardial surface and the visceral layer of the pericardium. While EAT is known to exert a protective cardiovascular effect^1^, it has also been associated with cardiovascular disease and myocardial function^2^. Cross-sectional clinical and translational studies have suggested that EAT can contribute to the progression of coronary artery disease (CAD), heart failure, and atrial fibrillation^3^. EAT releases biochemical factors such as adiponectin, nitric oxide, and methyl-ester prostacyclin, which can affect the myocardium and coronary vasculature through paracrine, vasocrine, or combined effects^4^. Expansion of EAT leads to increased peri-atrial fibrosis, while EAT inflammation can affect the coronary adventitia via the outside-to-inside pathway^4^. Therefore, assessments of EAT expansion (volume) and inflammation may provide prognostic information in patients with heart disease^3^. The volume or radiodensity of EAT can be non-invasively quantified and accurately measured by automatic quantitative measurement software using computed tomography (CT) images^5^. EAT radiodensity is reflected in terms of tissue attenuation and expressed as Hounsfield units (HU). Lipids are represented by negative HU values (from − 30 to − 190 HU) on CT, and inflammation can increase fat attenuation on CT by inhibiting adipogenesis in epicardial fat^6^.

Recent studies have reported that increased EAT thickness is associated with more profound cardiac hemodynamic derangements, such as greater elevation in cardiac filling pressure and more severe pulmonary hypertension^7^. Some evidence suggests that EAT plays an important role in cardiovascular risk assessment^8,9^: A prospective study including asymptomatic patients reported that EAT volume and radiodensity measured using CT are associated with major adverse cardiac events^10^. In addition, EAT radiodensity has been associated with adverse cardiovascular events independent of EAT volume^11^. Changes in the radiodensity of fatty tissue on CT may represent changes in the composition of adipose tissue such as angiogenesis and inflammation^12^, which can in turn lead to adipose tissue remodeling and fibrosis^13^. As EAT radiodensity represents tissue inflammation and cardiovascular risk^6^, this parameter may have clinical relevance in patients with end-stage renal disease (ESRD), given that inflammation is highly prevalent and linked to cardiovascular disease in this population^14^. Moreover, sudden cardiac death is the largest contributor to mortality among patients with ESRD^15^, highlighting the need to identify a new biomarker for cardiovascular disease in the ESRD population.

Most previous studies regarding EAT included non-ESRD populations and did not investigate long-term outcomes such as all-cause mortality^8,10^. While some studies have reported that high EAT volume increases the risk of cardiovascular events in patients with reduced kidney function^16^ and predicts mortality among patients undergoing incident hemodialysis^17^, the relationship of EAT volume and radiodensity with long-term morality in the context of hemodialysis remains elusive. Therefore, the present study aimed to investigate whether EAT radiodensity and volume are related to all-cause mortality in patients undergoing hemodialysis.

## Results

### Study population and clinical features

A total of 2,122 coronary CTAs were performed during study period. We excluded 1,857 patients without ESRD. After an in-depth review of coronary CTA data, four patients were excluded due to poor image quality; finally, 221 patients with ESRD were included in the analysis (Fig. S1). Among them, 82 deaths (37.1%) occurred during a median follow-up period of 29.63 months. Characteristics of the study population are presented in Table 1. The mean patient age was 64.88 ± 11.09 years, and 107 patients (48.42%) were men. Most patients (151, 69%) were taking antiplatelet agents. There were 53 cases (24%) of confirmed multi-vessel CAD following coronary CTA. After coronary CTA, 38 patients (17.19%) underwent subsequent invasive coronary angiography as they required further evaluation. Among them, 29 and three patients underwent PCI and CABG, respectively.

**Table 1:** Baseline characteristics of patients with ESRD according to all-cause mortality.

	Total (n = 221)	Non-survival (n = 82)	Survival (n = 139)	*p* value
Age (years)	64.88 ± 11.09	68.88 ± 9.68	62.52 ± 11.23	< 0.001
Male n, (%)	107 (48.42%)	41 (50%)	66 (47.48%)	0.824
BMI (kg/m^2^)	22.43 (19.93, 25.04)	22.35 (19.9, 25.06)	22.57 (20.27, 24.87)	0.803
EAT volume (mL)	126.83 (84.75, 179.69)	138.23 (92.8, 203.13)	124.34 (80.4, 166.25)	0.169
EAT radiodensity (HU)	− 73.72 ± 6.8	− 72.88 ± 7.02	− 74.22 ± 6.64	0.165
Hemodialysis duration, years	5 (1.6, 11.2)	5.15 (2.33, 12)	5 (1.5, 10.75)	0.332
**Cormorbidities**				
Diabetes	105 (47.51%)	47 (57.32%)	58 (41.73%)	0.036
Smoking	83 (37.56%)	35 (42.68%)	48 (34.53%)	0.287
Previous PCI or CABG state	37 (16.74%)	22 (26.83%)	15 (10.79%)	0.004
Baseline LVEF (%)	60 (48, 66)	57 (45, 65)	61 (50, 67)	0.029
**Relevant medications**				
Antiplatelet agent	151 (68.95%)	63 (76.83%)	88 (64.23%)	0.072
Aspirin	48 (21.92%)	23 (28.05%)	25 (18.25%)	
P2Y12 inhibitor	36 (16.44%)	10 (12.2%)	26 (18.98%)	
Cilostazol	3 (1.37%)	2 (2.44%)	1 (0.73%)	
Dual antiplatelet	64 (29.22%)	28 (34.15%)	36 (26.28%)	
Statin	120 (55.05%)	46 (56.1%)	74 (54.41%)	0.919
Beta-blocker	93 (42.66%)	39 (47.56%)	54 (39.71%)	0.320
ACE inhibitor or ARB	91 (41.74%)	33 (40.24%)	58 (42.65%)	0.836
Phosphate binder	169 (76.47%)	64 (78.05%)	105 (75.54%)	0.794
Calcium-free binder	46 (20.81%)	15 (18.29%)	31 (22.3%)	
Calcium-based binder	123 (55.66%)	49 (59.76%)	74 (53.24%)	
**Laboratory data**				
Albumin (g/dL)	3.94 ± 0.52	3.8 (3.42, 4.18)	4 (3.75, 4.3)	0.002
Total cholesterol (mg/dL)	137 (114.5, 162.5)	124 (109, 153)	142.5 (121, 167)	0.019
LDL cholesterol (mg/dL)	77 (56, 98)	70 (53.5, 92.5)	83.5 (63.75, 101.25)	0.031
Hemoglobin (g/dL)	10.46 ± 1.58	10.51 ± 1.59	10.43 ± 1.58	0.746
Calcium (mg/dL)	9.1 (8.5, 9.6)	9 (8.4, 9.6)	9.2 (8.6, 9.65)	0.113
Phosphorus (mg/dL)	4.61 ± 1.7	4.43 ± 1.81	4.71 ± 1.62	0.250
Calcium × phosphorus	40.67 (29.76, 51.33)	38.07 (27.13, 50.91)	41.85 (33.74, 51.86)	0.076
hs-CRP (mg/dL)	0.53 (0.12, 2.18)	0.87 (0.18, 3.37)	0.4 (0.1, 1.49)	0.026
**Result of coronary CTA**				
Multi-vessel CAD	53 (23.98%)	23 (28.05%)	30 (21.58%)	0.355
Left main disease	4 (1.81%)	2 (2.44%)	2 (1.44%)	0.629
Subsequent invasive CAG	38 (17.19%)	18 (21.95%)	20 (14.39%)	0.544
Subsequent PCI	29 (13.12%)	10 (12.2%)	19 (13.67%)	0.914
Subsequent CABG	3 (1.36%)	1 (1.22%)	2 (1.44%)	> 0.99
Duration of follow up (months)	29.63 (16.67, 44.7)	15.98 (7.9, 33.02)	33.87 (23.13, 50.48)	< 0.001

*ACE* angiotensin converting enzyme, *ARB* Angiotensin II receptor blocker, *BMI* body mass index, *CABG* coronary artery bypass graft, *CAD* coronary artery disease, *CTA* CT angiography, *EAT* epicardial adipose tissue, *ESRD* end-stage renal disease, *HDL* density lipoprotein, *hs-CRP* high sensitivity C-reactive protein, *HU* hounsfield unit, *LDL* low density lipoprotein, *LVEF* left ventricular ejection fraction, *MI* myocardial infarction, *PCI* percutaneous coronary intervention.

### Comparison of survivor and non-survivor groups

Non-survivors were older and had lower left ventricular ejection fraction and albumin levels than survivors. In addition, the frequency of diabetes, history of PCI or CABG, and increased high-sensitivity C-reactive protein (hs-CRP) levels was greater among non-survivors than among survivors. However, there were no significant differences in serum calcium, phosphate, or calcium × phosphate levels between the two groups. Furthermore, there were no significant differences in the use of cardiovascular disease-related medications such as anti-platelet agents, statins, or phosphate binders between the two groups. The proportion of revascularizations (PCI or CABG) performed after invasive coronary angiography was also similar in the two groups (Table 1).

### Relationship between EAT radiodensity/volume and other variables

EAT radiodensity among the study population exhibited a normal distribution (Fig. S2). Among the variables expected to affect EAT radiodensity or volume, the change in EAT radiodensity exhibited a moderate negative correlation with EAT volume (R =  − 0.5944) (Fig. S3) and body mass index (BMI) (R =  − 0.4521). EAT volume exhibited a moderate positive correlation with BMI (R = 0.5728). In the lipid profile, HDL cholesterol and triglyceride levels exhibited a slight negative correlation with EAT radiodensity (Table S1). The duration of dialysis was not correlated with EAT radiodensity or volume, and there were no differences in EAT radiodensity based on the presence or absence of diabetes mellitus (− 73.98 ± 6.59 HU vs. − 73.49 ± 7.00 HU; *p* = 0.59), or on the presence or absence of a history of coronary revascularization (PCI or CABG) (− 5.26 ± 6.96 vs. − 73.41 ± 6.74; *p* = 0.13). When comparing the three CAD categories, we observed no significant differences in EAT radiodensity between the groups (Fig. S4). EAT radiodensity was lower (− 74.75 ± 7.16 HU vs. − 72.41 ± 6.13 HU; *p* = 0.01) and volume was larger (155.48 ± 80.11 vs. 127.19 ± 74.1, *p* = 0.007) in statin-treated patients than in non-statin-treated patients.

### EAT radiodensity/volume in relation to mortality

We conducted Cox proportional-hazards regression analysis to identify independent predictors of all-cause mortality in patients with ESRD (Table S2). Fifteen variables were included in the analysis. After adjusting for confounding variables, the following were identified as significant independent predictors: (1) EAT radiodensity, (2) diabetes, (3) age, (4) duration of dialysis, and (5) hs-CRP level. Increased EAT radiodensity was associated with higher all-cause mortality in this analysis (HR: 1.055; 95% CI 1.015–1.095; *p* = 0.006). However, EAT volume was not significantly associated with all-cause mortality in either the univariable or multivariable analysis. After adjusting for confounding variables, the hazard ratio for mortality was still significant between the different models (Table 2).

**Table 2:** Association between EAT radiodensity and all-cause mortality in crude and multivariable adjusted cox regression analysis.

Model	Variables	Hazard ratio (95% CI)	*p* value
		EAT radiodensity	
1	Crude model	1.026 (0.994–1.060)	0.111
2	Model 1 + traditional risk factors*	1.055 (1.015–1.095)	0.006
3	Model 2 + BMI	1.055 (1.015–1.095)	0.006
4	Model 3 + HbA1c	1.057 (1.012–1.104)	0.013
5	Model 4 + high risk plaque	1.066 (1.023–1.112)	0.003

*BMI* body mass index, *CABG* coronary artery bypass graft, *CAD* coronary artery disease, *CT* computed tomography, *EAT* epicardial adipose tissue, *hs-CRP* high sensitivity C-reactive protein, *LVEF* left ventricular ejection fraction, *PCI* percutaneous coronary intervention.

*Adjusted for age, sex, diabetes status, smoking status, PCI or CABG (Previous or subsequent), multi-vessel CAD on CT, statin use, duration of dialysis, serum calcium x phosphorus, serum albumin, serum hemoglobin, serum hs-CRP, and LVEF.

**Figure 1:**
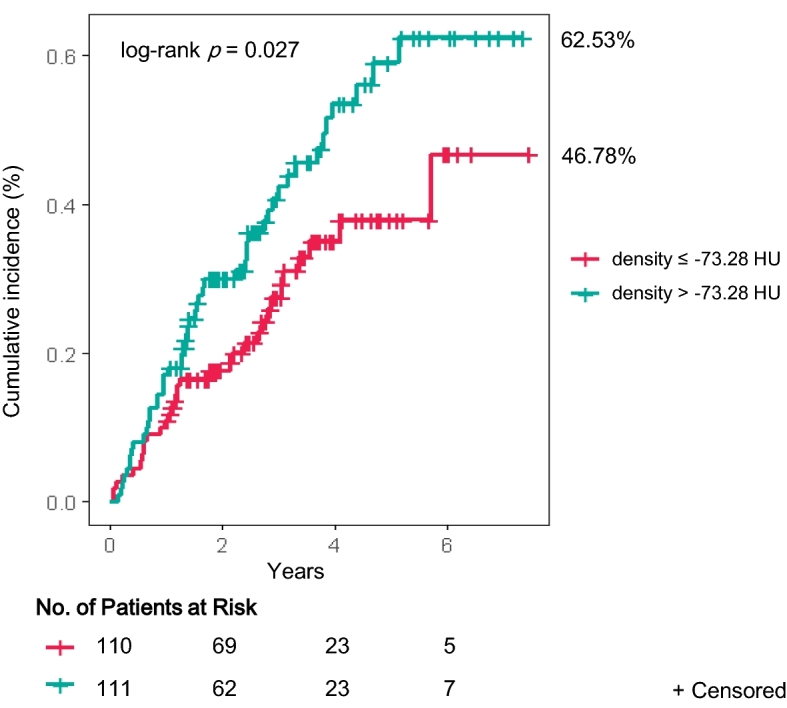
Comparison of the risks factors for all-cause mortality between the high and low EAT radiodensity groups. *EAT* epicardial adipose tissue, *HU* Hounsfield unit.

The optimum cutoff value of EAT radiodensity for predicting all-cause mortality derived from the receiver operating characteristic (ROC) curve analysis was − 73.28 HU. The probability of all-cause mortality in the ESRD population during the follow-up period is shown in Fig. 1. The mortality rate was significantly higher in the high EAT radiodensity group (> − 73.28 HU) than in the low EAT radiodensity group (62.53% vs. 46.78%, log-rank *p* = 0.027).

Cox proportional models further explored the prognostic value of EAT radiodensity to predict all-cause mortality over traditional risk factors (Table 3). Although there was a slight prognostic gain from adding EAT radiodensity to traditional risk factors, the overall discriminatory power did not differ substantially from the basic models (Comparison of ROC curves, p-value = 0.232) (Fig. S5).

**Table 3:** Prognostic value of EAT radiodensity in the prediction of all-cause mortality in addition to a basic set of traditional risk factors in patients with ESRD.

	Pseudo R^2^	AUC	− 2 log likelihood
Basic model^†^	0.1669	0.754 (0.679–0.830)	176.3983
Basic model with EAT radiodensity	0.1857	0.774 (0.700–0.848)	172.4072

*AUC* area under the curve, *BMI* body mass index, *CABG* coronary artery bypass graft, *CAD* coronary artery disease, *EAT* epicardial adipose tissue, *ESRD* end-stage renal disease, *hs-CRP* high sensitivity C-reactive protein, *LVEF* left ventricular ejection fraction, *PCI* percutaneous coronary intervention.

^†^Variables considered in the model were: age, sex, diabetes status, smoking status, PCI or CABG (Previous or subsequent), multi-vessel CAD on CT, statin use, duration of dialysis, serum calcium x phosphorus, serum albumin, serum hemoglobin, serum hs-CRP, and LVEF.

## Discussion

In this study, we investigated the ability of differences in EAT volume and radiodensity to predict mortality in patients with ESRD undergoing hemodialysis. Our findings indicated that higher EAT radiodensity was associated with a poorer prognosis after adjustment for confounding factors. However, EAT volume did not predict long-term mortality in patients undergoing prevalent hemodialysis. These findings support the notion that EAT has active biologic properties and suggest a potential role of EAT radiodensity as a biomarker for patients with ESRD.

EAT radiodensity is advantageous as a biomarker, as it is relatively simple to measure when compared with perivascular or pericoronary fat volume/density. Moreover, as EAT can be quantified from non-contrast CT^10^, there is no risk associated with exposure to contrast medium, which is especially critical for patients with chronic kidney disease. These benefits make determining EAT radiodensity a useful strategy for identifying high-risk patients with ESRD or renal insufficiency. Further studies involving larger cohorts of patients with ESRD are required to validate the applicability of EAT radiodensity on non-contrast CT.

Analyzing CTA imaging sequences may help to elucidate the biological effects of vascular inflammation such as expression of adipogenic genes, average adipocyte size, and lipid accumulation in pericoronary adipose tissue^6^. These findings suggest that high radiodensity in perivascular fat reflects prominent tissue inflammation. Considerable evidence suggests that EAT is closely related to coronary atherosclerosis^10,18–20^; however, in our study, the severity of CAD was not correlated with EAT radiodensity. As reported in another study^21^, it is possible that the degree of CAD was not accurately assessed due to the high calcium burden in patients with ESRD. Indeed, another study reported that calcium blooming artifacts are a major concern when assessing the degree of coronary artery stenosis^22^.

However, EAT radiodensity may reflect myocardial states other than coronary artery atherosclerosis. Many studies have reported that EAT abnormalities are associated with atrial fibrillation^3,23,24^, while another has noted that arrhythmogenic mechanisms may include adipocyte infiltration into the heart^25^. Given that sudden cardiac death is the leading cause of mortality among patients with ESRD^15^, additional studies are required to determine whether EAT radiodensity and fatal arrhythmias (e.g., ventricular tachycardia/fibrillation) are related.

It is well known that chronic inflammation is highly prevalent among patients with ESRD due to uremia-induced nutritional and catabolic alterations^26^, and the presence of inflammation has been associated with increased mortality risk in these patients^27^. Another study reported that inflammation, as determined based on higher CRP and interleukin (IL)-6 levels, was associated with a higher risk of sudden cardiac death among patients with ESRD^28^. Our results are consistent with those of a previous study^29^ in which the authors reported a significant association between CRP levels and mortality; however, EAT radiodensity was not associated with CRP levels. Therefore, we presumed that EAT radiodensity reflects a more heart-specific inflammatory state rather than systemic inflammation, and that this inflammatory response in the heart may contribute to mortality in patients with ESRD. As there is no fascia or other separating tissue, adipocytes in the epicardium may directly affect the myocardium, exacerbating myocardial lipotoxicity^30^. Myocardial lipotoxicity and extra-cardiac adiposity result in increased heart weight and decreased systolic function^31^, left ventricular hypertrophy, electrocardiographic abnormalities, and increased arrhythmogenicity^32^. We believe that chronic myocardial inflammatory conditions also influence EAT and contribute to mortality in patients with ESRD.

Our findings indicate that, unlike EAT radiodensity, EAT volume is not associated with all-cause mortality in patients undergoing maintenance hemodialysis. A previous study reported that high EAT volume predicts mortality in patients undergoing incident hemodialysis^17^. All patients in that study were using a phosphate binder, but only 76% were in our study. The difference in results is likely due to differences between the study populations. Previous studies have indicated that phosphate binders can increase EAT volume in patients undergoing hemodialysis^33^ and that high-phosphate treatments can also affect EAT volume in these patients. Therefore, we presumed that qualitative assessments of EAT based on inflammatory cell deposits and fibrosis are superior to quantitative assessments of EAT such as volume in terms of predicting prognosis in patients with ESRD.

Our study is subject to limitations. First, since our study was a retrospective study that involved a limited number of participants, there are limitations in showing direct causality between EAT density and mortality and generalizing the cutoff value for EAT density that predicts mortality. However, our mortality data from the Korean National Statistical Office data is very accurate. Therefore, the findings of our study may be considered robust despite the small sample size. Further large-scale prospective studies are needed to confirm this finding. Second, we were unable to assess CVD related outcomes in this study. However, several studies have reported the major cause of death in ESRD is CVD^15,34^. Third, despite coronary calcium score being an important predictor of cardiac events, we were unable to assess the extent of coronary calcification due to the CTA protocol of our institution and retrospective study design. Last, our study indeed showed that although the there was a slight prognostic gain to adding EAT radiodensity to traditional risk factors, the overall discriminatory power did not differ significantly.

In conclusion, Higher EAT radiodensity assessed by coronary CTA was associated with higher long-term all-cause mortality in patients with ESRD. Due to ease of measurement, EAT radiodensity may provide a useful method to noninvasively assess mortality risk in patients undergoing prevalent hemodialysis. Further prospective studies should need to confirm the cutoff value of EAT density that predicts the mortality.

## Methods

### Study population

This retrospective study was performed at a tertiary medical center. Between January 2012 and December 2018, a total of 2122 consecutive patients underwent coronary CT angiography (CTA) at our institution due to clinical indications such as chest discomfort or dyspnea. Among these patients, patients with ESRD were identified based on a review of medical records. All CTA scans of the patients finally included in this study were performed after initiation of dialysis. This study was carried out in accordance with the ethical principles of the Declaration of Helsinki and was approved by the Institutional Review Board of Soonchunhyang University Hospital (IRB no. 2019-04-032), which waived the requirement for informed consent due to the retrospective nature of the study.

### Variables and definitions

Risk factors and clinical diagnoses for the included patients at the time of coronary CTA were acquired from the electronic medical record system of our institution. Laboratory variables, such as serum lipid profile and hemoglobin level, were also collected. History of percutaneous coronary intervention (PCI) or coronary artery bypass grafting (CABG) was assessed based on a review of the medical records and the results of coronary CTA. Left ventricular ejection fraction was evaluated via echocardiography (modified Simpson’s method). The coronary CTA results were analyzed to identify multi-vessel CAD and left-main (LM) disease, which can also influence mortality. Multi-vessel CAD was defined as more than two major epicardial arteries with > 50% stenosis, while LM disease was defined as LM coronary stenosis > 50%. The severity of CAD was categorized as follows^35^: normal (Absence of plaque and no luminal stenosis) or mild CAD (plaque with 20–49% stenosis), one-vessel CAD (one major epicardial artery having > 50% stenosis), and multi-vessel CAD. Calcified plaque was defined as lesions with 50% or greater calcium^35^. We defined high-risk plaque features as the presence of spotty calcification, low-attenuation plaques, positive remodeling, or napkin-ring sign^36^. In select patients, invasive coronary angiography was performed after coronary CTA. The revascularization strategy after invasive coronary angiography (PCI or CABG) was also analyzed.

### Coronary CTA and fat quantification

All patients underwent CTA using two types of 128-slice multi-detector CT scanners (SOMATOM Definition Edge, Siemens Medical Solutions, Erlangen, Germany; and Discovery CT750 HD, GE Healthcare, Milwaukee, WI, USA). CT was performed using the following parameters: collimation, 0.6 mm; slice acquisition, 2 × 64 (or 128) × 0.6 mm by means of x-flying focal spot; pitch, 0.2; tube voltage, 100–120 kV; gantry rotation time, 330 ms. Patients received an 80-mL bolus of contrast medium. All coronary CTA datasets were acquired using retrospective electrocardiographic gating and were automatically selected to allow synchrony with the heartbeat to enable efficient adaptive multi-segment image reconstruction.

**Figure 2:**
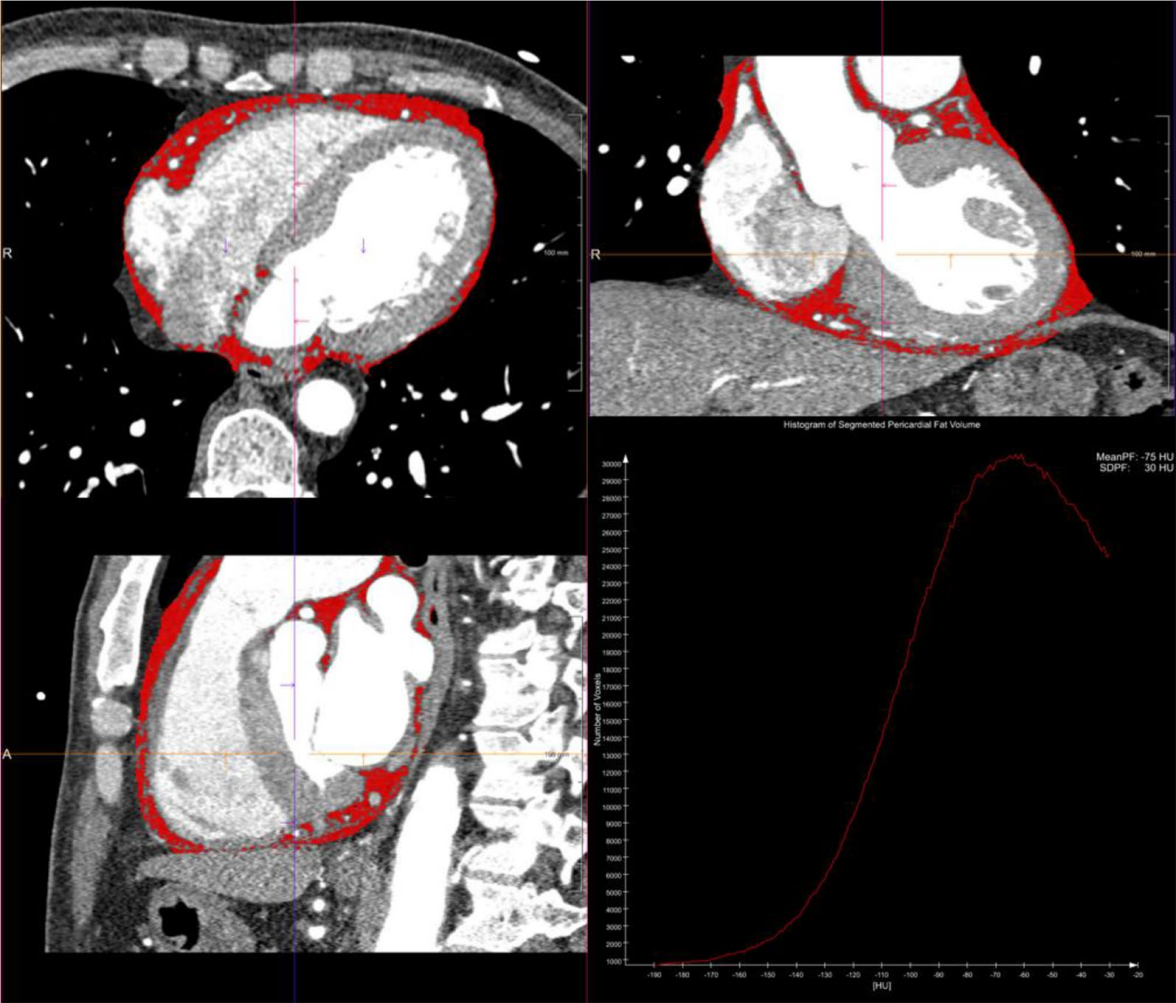
Epicardial adipose tissue (EAT) quantification. Measurement of EAT volume and radiodensity by automated software. EAT is highlighted in red.

CTA data were transferred to a post-processing workstation (syngo.via, Siemens) for further analysis by an investigator with 9 years of experience in cardiothoracic CT imaging. A dedicated semi-automatic software prototype (Cardiac Risk Assessment 1.2.1 syngo.via FRONTIER, Siemens AG, Healthcare Sector, Forchheim, Germany) was used for quantitative fat analysis. The EAT depot was defined as the fat tissue between the outer wall of the myocardium and the visceral layer of the pericardium. The software semi-automatically reconstructed the pericardium into a three-dimensional region of interest. Within this region, delineated by the pericardium, contiguous voxels between the limits of − 190 HU and − 30 HU were defined as adipose tissue. Radiodensity (mean and standard deviation with histogram) and volume were estimated across the total EAT (Fig. 2). Measurements were manually edited in areas affected by coronary calcification induced blooming effects, streaks, or motion artifacts if necessary. To adjust for the attenuation difference between scans performed at different tube voltages, the radiodensity for scans performed at 100 kVp was corrected by dividing by a conversion factor of 1.11485 to ensure that they could be compared to scans performed at 120 kVp^12,37^.

### Outcome

The main outcome of the present study was all-cause mortality. Mortality data were obtained from the Korean National Statistical Office (Microdata Integrated Service, on-demand, 20,180,619, https://mdis.kostat.go.kr), as it is mandatory to report the death of any Korean national to the National Statistical Office. The data included deaths among study participants occurring before December 31, 2019. Data regarding the cause of death were not used in further analyses, as the cause of death did not depend on an accurate medical assessment.

### Data analysis

Categorical variables are presented as numbers and percentages and were analyzed using chi-square or Fisher’s exact tests. Continuous variables with normal distributions are expressed as the mean ± standard deviation and were analyzed using independent Samples *t*-tests. Continuous variables without normal distributions were confirmed using the Shapiro–Wilk test, presented as the median (interquartile range), and analyzed using the Mann–Whitney U-test. When EAT radiodensity was analyzed according to the degree of CAD, statistical comparisons of individual groups were based on a one-way analysis of variance (ANOVA). Pearson’s correlation coefficients were calculated to determine the relationships between EAT radiodensity and other continuous variables.

All-cause mortality according to the degree of EAT radiodensity was analyzed using the Kaplan–Meier method and log-rank tests. In addition, multivariable stepwise Cox proportional-hazards regression analysis was used to identify independent predictors of all-cause mortality. Factors used in the multivariable analysis included those with *p* values < 0.10 in the univariable analysis as well as variables with known prognostic values. *p* < 0.05 was considered significant. We conducted prognostic models to determine whether EAT radiodensity adds clinically relevant prediction power to traditional risk factors. The explained variability is presented as the pseudo R^2^ from logistic regression models with all-cause mortality as the dependent variable and the − 2 log likelihood from Cox regression hazard models. The discriminatory power was calculated by area under the receiver operating characteristic curve analysis. All statistical analyses were performed using R version 3.6.1 and Rex version 3.0.3 (RexSoft Inc., Seoul, Korea).

## Supplementary Information

Supplementary Information.

## Data Availability

The data underlying this article cannot be shared publicly to protect the privacy of individuals that participated in the study. The data will be shared on reasonable request to the corresponding author.
