# Supplementary material for: Epicardial adipose tissue radiodensity is associated with all-cause mortality in patients undergoing hemodialysis
Source: Sci Rep. 2021 Nov 29;11:23090. doi: 10.1038/s41598-021-02427-4 (PMC8630096; doi:10.1038/s41598-021-02427-4)
Supplement: Supplementary file 1 — Supplementary Information. [file 41598_2021_2427_MOESM1_ESM.docx]

**Supplemental Materials**

Table S1. Correlation between the EAT radiodensity/volume and continuous covariates in study population.

|  | **Radiodensity** | | **Volume** | |
| --- | --- | --- | --- | --- |
|  | **R** | ***p* value** | **R** | ***p* value** |
| Age (years) | -0.0787 | 0.244 | 0.1841 | 0.006 |
| BMI (kg/m2) | -0.4521 | <0.001 | 0.5728 | <0.001 |
| BSA (m2) | -0.3212 | <0.001 | 0.4849 | <0.001 |
| Duration of dialysis | 0.0729 | 0.280 | 0.0522 | 0.440 |
| Total cholesterol (mg/dL) | -0.0848 | 0.243 | -0.0123 | 0.866 |
| LDL cholesterol (mg/dL) | -0.1256 | 0.090 | 0.0331 | 0.656 |
| HDL cholesterol (mg/dL) | -0.2329 | 0.005 | -0.2517 | 0.002 |
| Triglyceride (mg/dL) | -0.4244 | <0.001 | 0.2978 | <0.001 |

The R and *p* values for the correlation between the EAT radiodensity/volume and continuous covariates were calculated by Pearson’s correlation coefficient analysis. BMI, body mass index; BSA, body surface area; EAT, epicardial adipose tissue; eGFR, estimated glomerular filtration rate; HDL, high density lipoprotein; LDL, low density lipoprotein.

Table S2. Analysis of risk factors for long term all-cause mortality in patients with ESRD.

| Variable | Univariable analysis | | Multivariable analysis | |
| --- | --- | --- | --- | --- |
|  | Hazard Ratio (95% CI) | *p* value | Hazard Ratio (95% CI) | *p* value |
| Age | 1.046 (1.025 - 1.067) | < 0.001 | 1.072 (1.045 - 1.100) | < 0.001 |
| Male | 1.206 (0.782 - 1.859) | 0.398 |  |  |
| Diabetes | 1.729 (1.114 - 2.685) | 0.015 | 2.189 (1.246 - 3.845) | 0.006 |
| Smoking | 1.363 (0.880 - 2.112) | 0.166 |  |  |
| PCI or CABG (Previous or subsequent) | 1.611 (1.033 - 2.511) | 0.035 |  |  |
| Multi-vessel CAD on CT | 1.162 (0.718 - 1.882) | 0.541 |  |  |
| Statin user | 1.199 (0.772 – 1.863) | 0.418 |  |  |
| Duration of dialysis (years) | 1.016 (0.985 - 1.049) | 0.322 | 1.044 (1.005 - 1.084) | 0.028 |
| Serum calcium x phosphorus | 0.984 (0.970 - 0.998) | 0.028 |  |  |
| Serum albumin (g/dL) | 0.574 (0.374 - 0.881) | 0.011 |  |  |
| Serum hemoglobin (g/dL) | 1.043 (0.909 - 1.195) | 0.551 |  |  |
| Serum hs-CRP (mg/dL) | 1.039 (0.998 - 1.082) | 0.061 | 1.043 (1.002 - 1.085) | 0.040 |
| LVEF (%) | 0.981 (0.963 - 0.998) | 0.029 |  |  |
| EAT volume | 1.002 (0.999 - 1.005) | 0.072 |  |  |
| EAT radiodensity | 1.026 (0.994 - 1.060) | 0.111 | 1.055 (1.015 - 1.095) | 0.006 |

BMI, body mass index; CABG, coronary artery bypass graft; CAD, coronary artery disease; CT, computed tomography; EAT, epicardial adipose tissue; ESRD, end stage renal disease; hs-CRP, high sensitivity C-reactive protein; HU, hounsfield unit; LVEF, left ventricular ejection fraction; PCI, percutaneous coronary intervention.

Figure S1. Flow chart of the study population. eGFR; estimated glomerular filtrate rate.

Coronary CT angiography between January 2012 and December 2018 (n = 2122)

**Excluded** as following reasons

- non-dialysis CKD patients (n = 40)
- poor image quality (n = 4)

Study population (n = 221)

Patients with chronic kidney disease (CKD)

(n = 265)

**Excluded** patients with eGFR > 60 mL/min/1.73 m (n = 1857)

Figure S2. EAT radiodensity distribution (n = 221, Shapiro-Wilk *p* = 0.5206).

EAT, epicardial adipose tissue


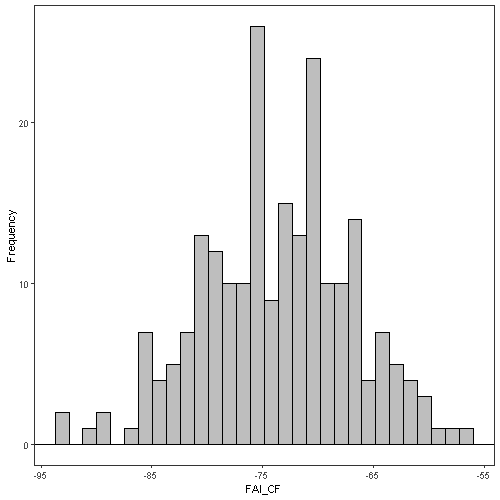


**Frequency (n)**

**EAT radiodensity (HU)**

Figure S3. Scatter plot of correlation between coronary CTA-derived EAT volume and radiodensity in study population (n=221). EAT volume and radiodensity show a modest negative correlation (R = -0.5944, *p* < .001).

CTA, CT angiography; EAT, epicardial adipose tissue


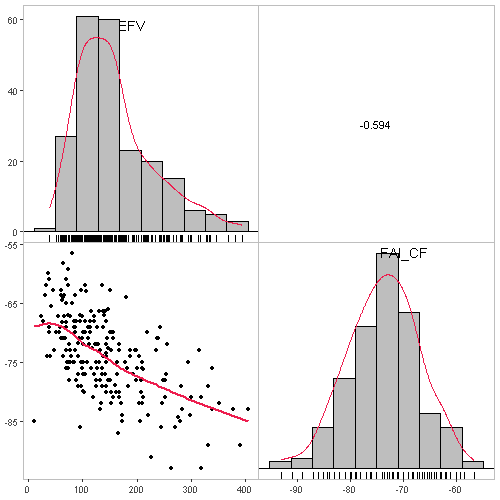


**R = -0.5944**

***p* < 0.001**

**EAT radio-density (HU)**

**EAT volume (mL)**

0

100

200

300

400

-85

-75

-65

-55

Figure S4. EAT radiodensity according to degree of CAD. The data are presented as arithmetic means and 95% CIs in an error bar plot. CAD, coronary artery disease; EAT, epicardial adipose tissue; SD, standard deviation.


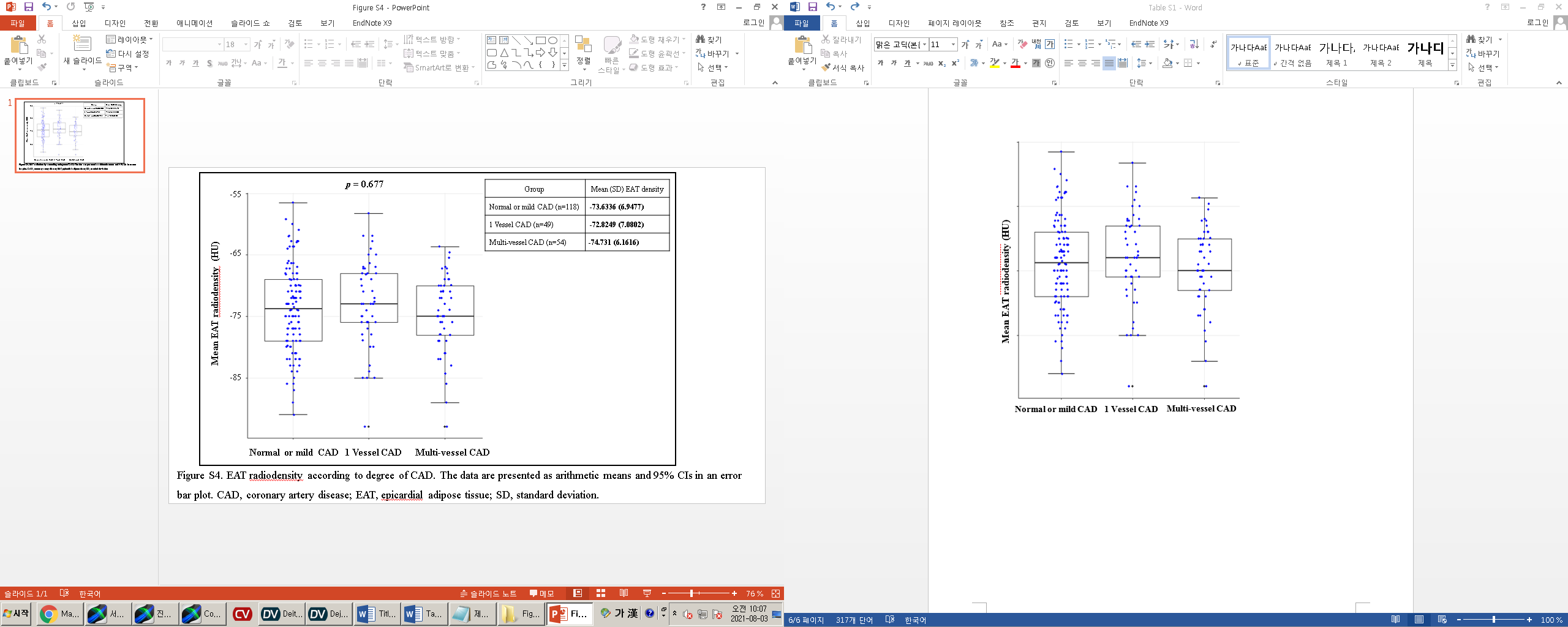


Figure S5. Comparison of area under curve (AUC) to predict the all-cause mortality.

**
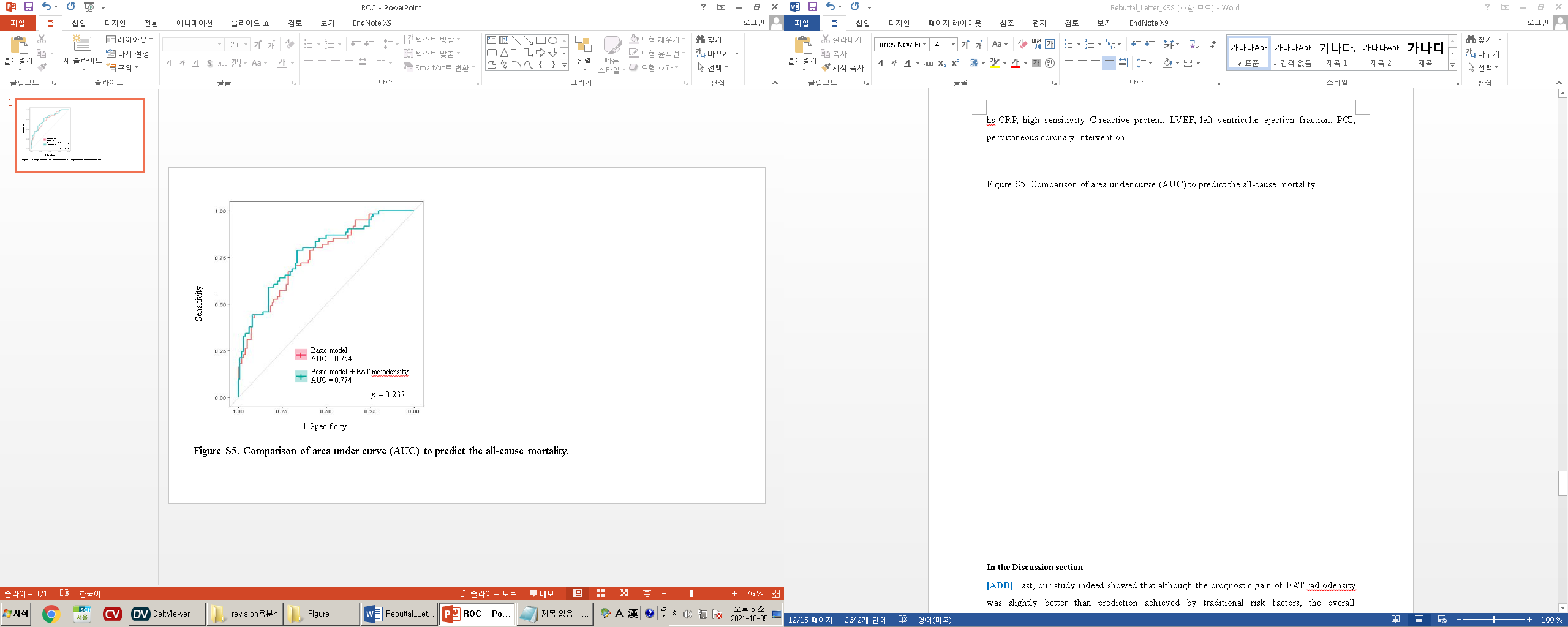
**
